# A novel therapeutic regimen based on the constrained-disorder principles may improve the response to dimethyl fumarate in multiple sclerosis: Findings from an exploratory feasibility open-label clinical trial

**DOI:** 10.1016/j.ibneur.2026.08.020

**Published:** 2026-09-02

**Authors:** Hillel Lehmann, Yoav Hershkowitz, Noa Hurvitz, Samuel Agus, Marc Berg, Yaron Ilan, Adi Vaknin-Dembinsky

**Affiliations:** a Department of Medicine, Hadassah Medical Center, Faculty of Medicine, Hebrew University, Jerusalem, Israel; b Oberon Sciences and Area 9 Innovation, USA; c Stanford University, Palo Alto, CA, USA; d Department of Neruology, Hadassah Medical Center, Faculty of Medicine, Hebrew University, Jerusalem, Israel

**Keywords:** Multiple sclerosis, Dimethyl fumarate, Artificial intelligence, Non-linearity, Variability

## Abstract

**Introduction:**

Dimethyl fumarate (DMF) is an oral disease-modifying therapy recommended for patients with relapsing-remitting multiple sclerosis (MS). However, a lack of efficacy, safety concerns, tolerability issues, and poor compliance often lead to discontinuation of DMF. In an innovative approach, this exploratory proof-of-concept clinical trial aimed to assess the feasibility and safety of introducing variability into treatment regimens to overcome compensatory mechanisms that underlie the loss of drug effectiveness and to generate preliminary signals of potential improvements in response to DMF.

**Methods:**

Seven MS patients treated with DMF were enrolled in an open-label, uncontrolled feasibility clinical trial in which an app provided a personalized therapeutic regimen, resulting in variability in dosages and administration times within predefined ranges. Prespecified feasibility endpoints included: (1) successful app installation and use; (2) patient adherence to the algorithmic dosing schedule; (3) completion of the 12-week follow-up; and (4) absence of serious adverse events. Exploratory outcome measures included the Expanded Disability Status Scale (EDSS), neurofilament light chain (NfL) levels, and MRI, which were assessed only as hypothesis-generating signals.

**Results:**

Although this study is a small exploratory proof of concept, the data suggest that AI-assisted personalized treatment is feasible and could improve clinical responses to DMF. The EDSS score improved in 2 patients (29%, p = 0.371), and the MRI remained stable in 6 patients (100%). These findings did not reach statistical significance and should be interpreted as exploratory only. A high patient engagement rate with the app was recorded during the study. No serious adverse events were observed.

**Summary:**

The results of this feasibility trial support the safety and feasibility of using a personalized algorithm that randomizes DMF regimens and provide preliminary hypothesis-generating signal data regarding potential improvements in therapy response. These findings are insufficient to support conclusions about efficacy. Controlled studies are needed to confirm these findings. (NCT06385197)

## Introduction

Dimethyl fumarate (DMF) is an oral disease-modifying therapy (DMT) recommended as first-line therapy for relapsing-remitting (RR) multiple sclerosis (MS) patients (Montes Diaz et al., 2018). DMF exhibits neuroprotective and immunomodulatory effects, along with a favorable benefit-to-risk profile. It remains an effective treatment for MS, with a favorable safety profile demonstrated over ten years of clinical use (Gold et al., 2020, Chinea et al., 2020). DMF is effective and safe for treating MS in real-world audits. Treatment-naive patients see significant benefits from DMF, and it also reduces the annualized relapse rate (ARR) in patients who switch from injectable therapies due to issues with tolerability and efficacy (Barros et al., 2020).

Lack of efficacy (50%), safety concerns (30%), tolerability issues (7%), and poor compliance (7%) are the reasons for DMF discontinuation over time. Higher pre-treatment EDSS is associated with DMF discontinuation (Lanzillo et al., 2020). Patients discontinued DMF due to side effects in 22% of cases, with gastrointestinal discomfort (12.7%) and lymphopenia (5.3%) being the most frequently reported (Miclea et al., 2016). In most cases, loss of response to DMF leads to replacing the treatment with other immunomodulatory medications that are not necessarily better or safer, some of which carry high side effects.

Second-generation artificial intelligence (AI) systems use variability-based algorithms to create personalized treatment plans by optimizing dosages and administration times. These systems prioritize clinical outcomes as endpoints for their algorithms. They dynamically adjust their outputs based on ongoing assessments of patient responses and adhere to the n = 1 concept, which focuses on individual subjects (Bayatra et al., 2024, Ilan, 2020a, Ilan, 2021a, Ilan, 2024). The platform addresses the compensatory mechanisms underlying the decreased effectiveness of chronic medications (Ilan, 2020a, Ilan, 2021b, Ilan, 2022a). The platform is founded on the Constrained Disorder Principle (CDP), which characterizes biological systems by their variability within dynamic boundaries. According to the CDP, malfunctions in biological systems occur either when inherent variability is lost or when variability exceeds established biological limits. Additionally, a second-generation AI system aids patients through an app that manages their medication schedules and dosages (Ilan, 2021b, Ilan, 2022b).

In the context of DMF, its primary mechanisms—Nrf2 pathway activation and modulation of immune cell trafficking via lymphocyte subtype shifts—may be subject to regulatory adaptation with fixed chronic dosing, providing a mechanistic rationale for introducing dosing variability (Hammer et al., 2018). However, direct MS-specific evidence for this approach remains to be established.

This open-label feasibility clinical trial aimed to assess the feasibility, safety, and tolerability of second-generation AI-regulated treatment regimens in potentially enhancing the response to DMF, and to generate preliminary exploratory data on clinical and biological signals to inform future controlled trials.

## Methods

### Study design

The clinical trial was a 12-week, open-label, uncontrolled, prospective feasibility study conducted at a single center. The primary purpose was to assess the feasibility, safety, and tolerability of the intervention. All clinical outcome measures (EDSS, NfL, MRI) were prespecified as exploratory signals only and were not powered to infer efficacy. Participants were recruited for the trial at Hadassah-Hebrew University Medical Center in Jerusalem, Israel. The Hadassah Medical Center IRB and the Israeli Ministry of Health (MOH_2022–07–07_011892) approved the trial. The trial was registered at ClinicalTrials.gov: NCT06385197.

## Participant flow

Of 128 screened patients, 121 were excluded for the following reasons: 68 did not meet the inclusion criteria (including the smartphone requirement and continuous DMF use ≥6 months without interruption); 34 declined to participate; and 19 were excluded for other reasons (comorbidities, inability to attend visits). Seven patients were enrolled and analyzed.

### Study population

Eligible subjects included those aged 18–75 years, male and female, diagnosed with MS.

### Inclusion and exclusion criteria

The study included adult non-pregnant patients diagnosed with multiple sclerosis (MS) and treated with dimethyl fumarate (DMF) without any other immunosuppressive treatment or steroid use for at least six months. Patients with severe infectious, malignant, autoimmune, or other systemic diseases were excluded from the study. Additionally, patients who could not provide written informed consent, lacked a smartphone, or failed to adhere to the visit schedule and protocol were excluded.

### Second-generation AI system

Altus Care™ is a mobile application developed by Area9 Innovation Apps (version 2.1). The system used in this study operated in Level 1 (open-loop) mode, in which the algorithm applies fully randomized dosing and timing within physician-defined ranges, without adaptive feedback from clinical outcomes during the trial. The permitted DMF dose range was the approved therapeutic range (120–480 mg/day) as specified by the treating neurologist for each patient. Administration times were randomized within a clinician-specified morning and/or evening window. In the event of a missed dose, the app sent a re-notification within 30 min; if unacknowledged, the dose was skipped per protocol. Clinicians retained full override authority and could modify dose ranges at any study visit. The system inputs were the physician-defined ranges; the outputs were daily push notifications to the patient specifying dose and timing. No dose exceeded what the patient was already receiving before enrollment, and no administration was more frequent than the pre-enrollment schedule.

## Feasibility endpoints (Prespecified)

The following feasibility endpoints were prespecified:

Successful installation and use of the Altus Care™ app
Patient adherence to the algorithmic dosing schedule (target: ≥80% of doses taken as notified)
Completion of the 12-week follow-up period
Absence of serious adverse events attributable to the variable dosing regimen

## Follow-up parameters

Throughout the follow-up period, the research coordinator made weekly phone calls to check clinical well-being and adherence. Physical examination and EDSS were assessed at weeks 6 and 12. NfL levels were measured before and after the intervention (Simoa pTau-181 Advantage v2.1 HD-1/JD-X kit, Quanterix). MRI was performed on a 3 T scanner (Philips Ingenia) at Hadassah Medical Center using a standardized protocol including T1, T2, FLAIR, and post-gadolinium T1 sequences. The baseline MRI was the most recent scan available within 6 months before enrollment. A follow-up MRI was performed within 12 months of study completion. Lesion assessment was performed by a board-certified neuroradiologist blinded to clinical data. Volumetric analysis was performed using Slicer 3D software. Safety monitoring included a complete blood count and hepatic function tests at baseline and at study end.

Note: All clinical outcome measures were exploratory. The study was not powered to detect differences in efficacy. The contributions of variable dosing and adherence support cannot be disentangled in this study design, as all patients received both the algorithmic variability intervention and app reminders, as well as weekly contact with a coordinator.

### Statistics

The Wilcoxon signed-rank test was used to analyze paired, continuous, non-parametric data.

## Results

### Subjects' demographics and safety measures

Table 1 presents the demographics of the patients who participated in the study. Seven patients were enrolled, two males and five females, with an average age of 37. All patients completed the 12-week follow-up period without experiencing any significant adverse events.

**Table 1:** Baseline characteristics.

Age, median (range)		37 (28−54)
Male, number (%)		2 (29)
Time since diagnosis of MS, median (range), Y		5 (3−21)
Time since DMF treatment, median (range) M		51.5 (24–87.2)
Concomitant diseases	Radiculopathy n (%)	1 (7)
	Migraine (%)	1 (7)

## Safety data

Lymphocyte counts were measured at baseline and study end. All patients maintained lymphocyte counts above 0.5 × 10⁹/L throughout the study. No serious infections were recorded. Gastrointestinal adverse effects (nausea, diarrhea, abdominal pain) were reported in 2/7 patients (29%) and were mild and self-limiting. Flushing was reported in 14% of patients (1/7). No treatment interruptions or discontinuations occurred. CBC and hepatic function tests were within normal limits at all timepoints. Safety data are presented in Supplementary Table S1.

## Adding variability in administration times may improve the clinical outcome of MS patients

### Exploratory clinical outcomes

**Fig. 1:**
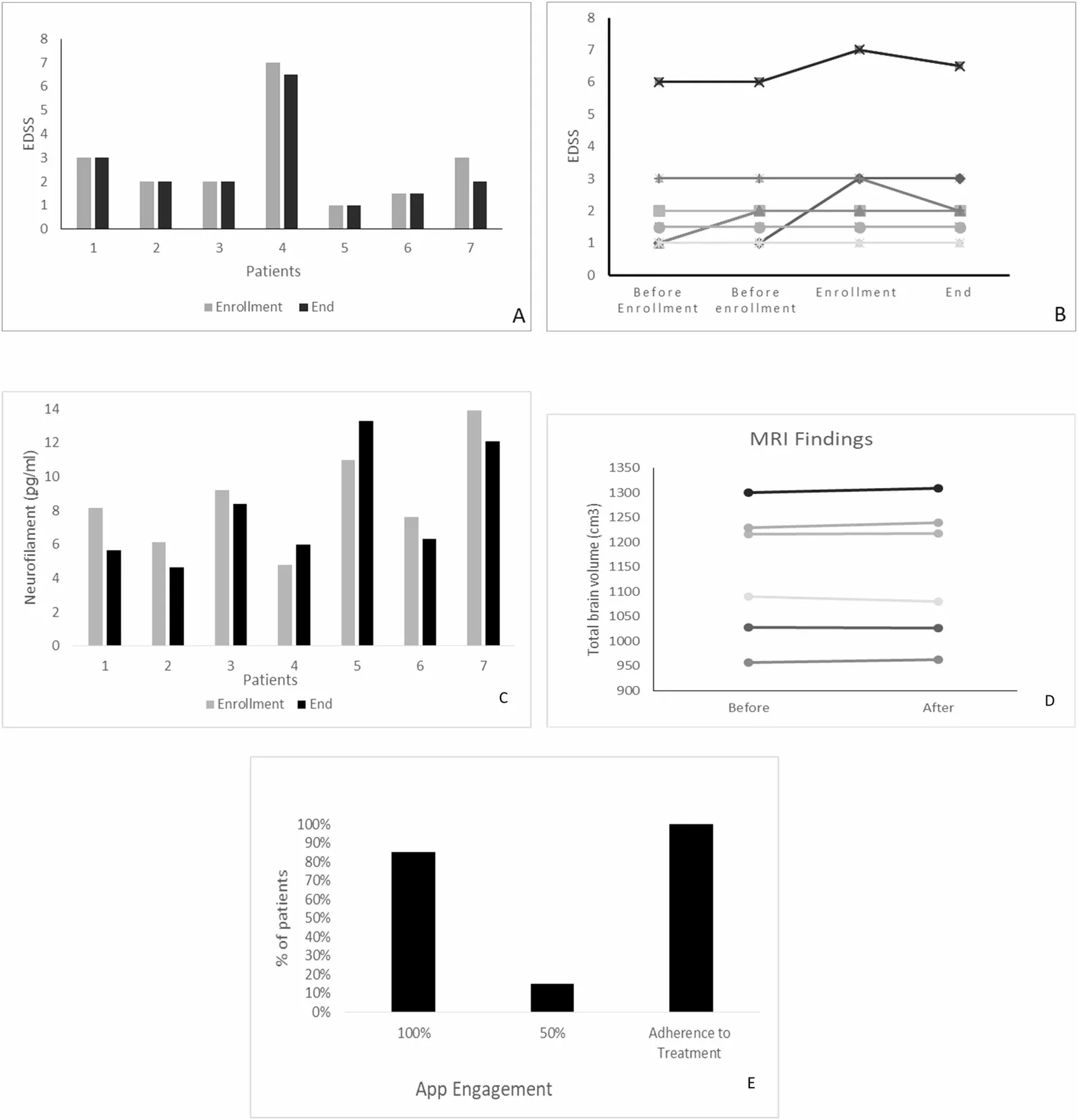
A: The effect of the intervention on the EDSS score was measured in all patients before and after the intervention. Each bar represents an individual patient before and after the intervention. 1B: Effect of intervention on disease progression. EDSS during the intervention was compared with its pre-intervention progression. Each line represents an individual patient. 1 C: The effect of the intervention on neurofilament light chains was measured in all patients before and after the intervention. Each bar represents an individual patient before and after the intervention. 1D: Effect of MRI intervention. Following the intervention, MRI was available in 6 patients. No change was noted in 6 patients (100%) compared with pre-intervention MRI. Volumetric analysis also demonstrated stability, with an average delta of 2.06 cm ^3^. Each line represents an individual patient. 1E: Percentage of patients who adhere to treatment regimens and use the app daily.

The second-generation, AI-based, personalized regimen was feasible and well-tolerated. The following data represent exploratory signals and should not be interpreted as evidence of efficacy. The EDSS score improved in 2 patients (29%, p = 0.371) by a mean of 0.5 points, and did not change in 5 patients (71%). No patients had a worsening of their EDSS score (Fig. 1A). These results were not statistically significant and are reported only as directional signals.

NfL levels decreased in 5/7 patients (71%, p = 0.375), with an average reduction of 1.5 pg/mL (Fig. 1C). This did not reach statistical significance. Six patients had MRIs available following the intervention. Compared with the pre-intervention MRI, no new T2- or gadolinium-enhancing lesions were noted in any of the six patients, and volumetric analysis showed stability, with an average delta of 2.06 cm³ (Fig. 1D).

Six patients used the app daily, as indicated by phone questionnaires. The seventh patient did not use the app daily but still adhered to the prescribed regimen. All patients adhered to the treatment regimens as indicated by the push notifications (Fig. 1E). These results indicate that the prespecified feasibility endpoints were met.

## Discussion

This open-label, exploratory, proof-of-concept feasibility clinical trial involving a small number of patients suggests that randomizing DMF regimens based on a second-generation personalized AI algorithm is feasible, safe, and may be associated with a preliminary signal of potential benefit. The data indicated that 29% of participants showed improvement in EDSS scores within 12 weeks, and all available MRIs demonstrated stable findings. These results are exploratory and insufficient to support conclusions about efficacy.

The constrained disorder principle (CDP) asserts that biological systems exhibit a degree of disorder within defined limits, and that variability is a hallmark of normal biological function (Kenig et al., 2021a, Azmanov et al., 2021, Potruch et al., 2020, Isahy and Ilan, 2021, Khoury and Ilan, 2019a, Khoury and Ilan, 2021, Kenig and Ilan, 2019, Ilan, 2019a, Ilan, 2019a, Gelman et al., 2020, Ishay et al., 2021b). In the context of DMF pharmacology, chronic fixed-dose administration may lead to regulatory adaptation through mechanisms such as normalization of lymphocyte subsets and desensitization of receptor pathways. The introduction of variability in dosing and timing is hypothesized to counteract these compensatory mechanisms. However, we emphasize that this rationale remains speculative in the context of MS, and direct mechanistic evidence specific to DMF and MS biology has not yet been established. The present study was not designed to test this mechanistic hypothesis.

Many patients with chronic diseases experience a loss of effectiveness from their treatments over time (Ilan, 2021a, Gelman et al., 2020, Ilan, 2019b, Ilan, 2019c, Ilan, 2020a, Ilan, 2020b, El-Haj et al., 2019, Ilan, 2019a, Ilan, 2019b, Ilan-Ber and Ilan, 2019, Forkosh et al., 2020, Shabat et al., 2021, Mietus et al., 2002, Navaneethakrishna and Manuskandan, 2021, Costa and Goldberger, 2019, Hammerle et al., 2020, Sen and McGill, 2018, Schutte et al., 2022, van den Bosch et al., 2021, Boripuntakul et al., 2022, Genon et al., 2022). Regular dosing can lead to decreased response and the development of drug tolerance. Utilizing algorithms based on variability may help address the body's compensatory responses to chronic medications (Ilan, 2021a, Kessler et al., 2020, Ishay et al., 2021a, Kolben et al., 2021, Kenig et al., 2021a, Azmanov et al., 2021, Potruch et al., 2020, Isahy and Ilan, 2021, Khoury and Ilan, 2019a, Khoury and Ilan, 2021, Kenig and Ilan, 2019, Ilan, 2019a, Ilan, 2019a, Gelman et al., 2020, Ishay et al., 2021b, Ilan and Spigelman, 2020, Hurvitz et al., 2021, Gelman et al., 2022, Azmanov et al., 2022, Hurvitz et al., 2022, Ilan, 2020b). CDP-based second-generation AI algorithms implement variability signatures in a personalized manner. These systems utilize a dynamic algorithm that continuously adjusts treatment plans based on important clinical outcomes. The algorithm considers various input factors, including the host's characteristics, the disease, and environmental conditions. It is designed to introduce variations in dosages and administration times of chronic medications to counteract the potential loss of drug effectiveness, ensuring a sustained therapeutic impact (Kessler et al., 2020, Ishay et al., 2021a, Kolben et al., 2021, Kenig et al., 2021a, Azmanov et al., 2021, Potruch et al., 2020, Isahy and Ilan, 2021, Khoury and Ilan, 2019a, Khoury and Ilan, 2021, Kenig and Ilan, 2019, Ilan, 2019a, Ilan, 2019a, Gelman et al., 2020, Ishay et al., 2021b, Ilan and Spigelman, 2020, Ilan, 2021b, Ilan, 2020b, Sigawi et al., 2023, Gelman et al., 2023, Hurvitz et al., 2024, Sigawi et al., 2024, Hurvitz et al., 2025). CDP-based second-generation AI algorithms implement variability signatures in a personalized manner. The algorithm considers various input factors, including the patient's characteristics, the disease, and the treating physician's defined ranges, and introduces variations in dosages and administration times within clinically approved limits.

The constrained disorder principle (CDP) asserts that biological systems display a certain degree of disorder within defined limits. Improper regulation or malfunction of these boundaries can result in biological dysfunction and disease. Variability is a hallmark of biological systems, indicating the proper function of the genome, organelles, and entire organs (Ilan, 2019a, Ilan, 2019b, Ilan, 2019c, Ilan, 2020a, Ilan, 2020b, El-Haj et al., 2019, Ilan, 2019a, Ilan, 2019b, Ilan-Ber and Ilan, 2019, Forkosh et al., 2020, Shabat et al., 2021). Heart rate variability and fragmentation are intrinsic characteristics of the heart, with reduced variability associated with higher cardiac mortality. Variability is also characteristic of normal breathing, blood pressure, brain function, and gait (Mietus et al., 2002, Navaneethakrishna and Manuskandan, 2021, Costa and Goldberger, 2019, Hammerle et al., 2020, Sen and McGill, 2018, Hurvitz et al., 2021, Schutte et al., 2022, van den Bosch et al., 2021, Boripuntakul et al., 2022, Genon et al., 2022, Saha and Baumert, 2019, Crawford et al., 2022).

A second-generation AI system manages the administration of the digital pill regimen, which consists of three levels. At the first level, a fully randomized approach is applied within predefined ranges established in the current trial. Physicians determine these ranges for DMF (dimethyl fumarate) dosages and administration times. Patients receive randomized reminders on their app regarding DMF dosages and administration times, all within clinically proven and regulatory-approved limits. This system operates in an open-loop mode, meaning it functions independently of specific inputs and outputs (Ilan, 2020a, Ilan, 2021b, Ilan, 2022a, Sigawi et al., 2023, Ilan, 2022c, Hurvitz and Ilan, 2023, Sigawi and Ilan, 2023).

The second level of the digital pill features a closed-loop system that dynamically adjusts variability ranges based on specific outcome measures. This algorithm takes into account the individual user's responses to customize the variability accordingly. At the third level, the algorithm integrates chronotherapy and considers factors such as heart rate variability, EEG variability, and variability in cytokine secretion (Bayatra et al., 2024, Ilan, 2021b).

Multiple mechanisms contribute to the reduced effectiveness of immunomodulatory agents, posing a challenge for overcoming and improving the response (Neuhaus et al., 2003, Khoury and Ilan, 2019b, Kalden and Schulze-Koops, 2017, Souto et al., 2016, Fine et al., 2019, Oleen-Burkey et al., 2012). These include genetic diseases, and environmental factors. Many MS patients who receive immunomodulatory agents discontinue them over several years. It is often due to insufficient clinical response or adverse effects (Yang et al., 2022).

The algorithm outlined in this study offers a way to enhance the clinical response to immunomodulatory drugs, regardless of the underlying mechanisms involved (Ilan, 2020a, Ilan, 2021b, Ilan, 2022a, Sigawi et al., 2023, Ilan, 2022c, Hurvitz and Ilan, 2023, Sigawi and Ilan, 2023). When administered early in the disease process, this approach could also help prevent resistance in patients with multiple sclerosis (MS) and other immune-mediated disorders. Its simplicity enables implementation for patients with chronic conditions who have either experienced reduced effectiveness from their current medications or have not fully responded to their treatment regimens (Ilan, 2021a, Ilan, 2022a, Kessler et al., 2020, Ishay et al., 2021a, Kolben et al., 2021, Kenig et al., 2021a, Azmanov et al., 2021, Potruch et al., 2020, Isahy and Ilan, 2021, Khoury and Ilan, 2019a, Khoury and Ilan, 2021, Kenig and Ilan, 2019, Ilan, 2019a, Ilan, 2019a, Gelman et al., 2020, Ishay et al., 2021b, Ilan and Spigelman, 2020, Hurvitz et al., 2021, Gelman et al., 2022, Azmanov et al., 2022, Kenig et al., 2021b).

## Limitations

The current study has several important limitations. First, the sample size of seven patients from a single center severely limits generalizability. Second, the open-label, non-randomized design with no concurrent control group precludes any inference regarding efficacy or noninferiority. Third, the 12-week follow-up period is insufficient to assess long-term outcomes relevant to MS, including relapse rate, disability progression, or MRI lesion accumulation. Fourth, and critically, the study design cannot disentangle the effect of algorithmic dosing variability from the effect of enhanced adherence support, as all patients received both app reminders and weekly coordinator contact, either of which may independently improve adherence and outcomes. Future controlled trials should include an active comparator arm in which participants receive a fixed-dose reminder app with no dosing variability and equivalent coordinator contact to isolate the specific contribution of the variability algorithm. Fifth, the outcome measures used (EDSS, NfL, MRI without gadolinium-enhancing lesion protocol emphasis) are not the most sensitive or standard endpoints for an MS intervention study; future studies should incorporate annualized relapse rate, steroid-treated relapses, new or enlarging T2 lesions, gadolinium-enhancing lesions, and validated cognitive and functional measures.

## Summary

The data from this exploratory feasibility study support the safety and feasibility of using a personalized AI algorithm to guide the randomization of DMF regimens and provide hypothesis-generating signal data that may inform future controlled trials. These findings are not sufficient to establish efficacy. Extensive prospective, randomized, controlled studies with appropriate comparator arms and robust MS-specific outcome measures are needed to confirm or refute these preliminary observations.

## Authors' contributions

HL, YH, and NH conducted the trial and analyzed the data; SA MB supported the app; AD and YI conceptualized it; and all authors reviewed the final version.

## CRediT authorship contribution statement

**Adi Vaknin-Dembinsky:** Writing – review & editing, Writing – original draft, Formal analysis, Conceptualization. **Noa Hurvitz:** Writing – review & editing, Writing – original draft, Formal analysis, Data curation. **Marc Berg:** Writing – review & editing, Writing – original draft, Conceptualization. **Yoav Hershkowitz:** Writing – review & editing, Writing – original draft, Investigation, Formal analysis, Data curation. **Hillel Lehmann:** Writing – review & editing, Writing – original draft, Investigation, Formal analysis, Data curation. **Samuel Agus:** Writing – review & editing, Writing – original draft. **Yaron Ilan:** Writing – review & editing, Writing – original draft.

## Disclosure

YI is the founder of Oberon Sciences. SA is a consultant for Oberon Sciences. MB is a consultant of Area9.

## Funding

NA.

## References

[bib1] Azmanov H., Bayatra A., Ilan Y. (2022). Digital analgesic comprising a second-generation digital health system: increasing effectiveness by optimizing the dosing and minimizing side effects. J. Pain. Res..

[bib2] Azmanov H., Ross E.L., Ilan Y. (2021). Establishment of an individualized chronotherapy, autonomic nervous system, and variability-based dynamic platform for overcoming the loss of response to analgesics. Pain. Physician.

[bib3] Barros A., Sequeira J., de Sousa A. (2020). Real-word effectiveness and safety of dimethyl fumarate in a multiple sclerosis portuguese population. Clin. Neuropharmacol..

[bib4] Bayatra A., Nasserat R., Ilan Y. (2024). Overcoming low adherence to chronic medications by improving their effectiveness using a personalized second-generation digital system. Curr. Pharm. Biotechnol..

[bib5] Boripuntakul S., Kamnardsiri T., Lord S.R. (2022). Gait variability during abrupt slow and fast speed transitions in older adults with mild cognitive impairment. PLoS. One.

[bib6] Chinea A., Amezcua L., Vargas W. (2020). Real-world safety and effectiveness of dimethyl fumarate in Hispanic or Latino Patients with Multiple Sclerosis: 3-year results from ESTEEM. Neurol. Ther..

[bib7] Costa M.D., Goldberger A.L. (2019). Heart rate fragmentation: using cardiac pacemaker dynamics to probe the pace of biological aging. Am. J. Physiol. Heart Circ. Physiol..

[bib8] Crawford L., Mills E., Meylakh N. (2022). Brain activity changes associated with pain perception variability. Cereb. Cortex.

[bib9] van den Bosch O.F.C., Alvarez-Jimenez R., de Grooth H.J. (2021). Breathing variability-implications for anaesthesiology and intensive care. Crit. Care.

[bib10] El-Haj M., Kanovitch D., Ilan Y. (2019). Personalized inherent randomness of the immune system is manifested by an individualized response to immune triggers and immunomodulatory therapies: a novel platform for designing personalized immunotherapies. Immunol. Res..

[bib11] Fine S., Papamichael K., Cheifetz A.S. (2019). Etiology and management of lack or loss of response to anti-tumor necrosis factor therapy in patients with inflammatory bowel disease. Gastroenterol. Hepatol. (N. Y).

[bib12] Forkosh E., Kenig A., Ilan Y. (2020). Introducing variability in targeting the microtubules: review of current mechanisms and future directions in colchicine therapy. Pharmacol. Res. Perspect..

[bib13] Gelman R., Bayatra A., Kessler A. (2020). Targeting SARS-CoV-2 receptors as a means for reducing infectivity and improving antiviral and immune response: an algorithm-based method for overcoming resistance to antiviral agents. Emerg. Microbes Infect..

[bib14] Gelman R., Berg M., Ilan Y. (2022). A subject-tailored variability-based platform for overcoming the plateau effect in sports training: a narrative review. Int. J. Environ. Res. Public. Health.

[bib15] Gelman R., Hurvitz N., Nesserat R. (2023). A second-generation artificial intelligence-based therapeutic regimen improves diuretic resistance in heart failure: results of a feasibility open-labeled clinical trial. Biomed. & Pharmacother..

[bib16] Genon S., Eickhoff S.B., Kharabian S. (2022). Linking interindividual variability in brain structure to behaviour. Nat. Rev. Neurosci..

[bib17] Gold R., Arnold D.L., Bar-Or A. (2020). Safety and efficacy of delayed-release dimethyl fumarate in patients with relapsing-remitting multiple sclerosis: 9 years' follow-up of DEFINE, CONFIRM, and ENDORSE. Ther. Adv. Neurol. Disord..

[bib18] Hammer A., Waschbisch A., Kuhbandner K. (2018). The NRF2 pathway as potential biomarker for dimethyl fumarate treatment in multiple sclerosis. Ann. Clin. Transl. Neurol..

[bib19] Hammerle P., Eick C., Blum S. (2020). Heart Rate Variability Triangular Index as a Predictor of Cardiovascular Mortality in Patients With Atrial Fibrillation. J. Am. Heart Assoc..

[bib20] Hurvitz N., Azmanov H., Kesler A. (2021). Establishing a second-generation artificial intelligence-based system for improving diagnosis, treatment, and monitoring of patients with rare diseases. Eur. J. Hum. Genet..

[bib21] Hurvitz N., Dinur T., Revel-Vilk S. (2024). A feasibility open-labeled clinical trial using a second-generation artificial-intelligence-based therapeutic regimen in patients with gaucher disease treated with enzyme replacement therapy. J. Clin. Med..

[bib22] Hurvitz N., Elkhateeb N., Sigawi T. (2022). Improving the effectiveness of anti-aging modalities by using the constrained disorder principle-based management algorithms. Front. Aging.

[bib23] Hurvitz N., Ilan Y. (2023). The constrained-disorder principle assists in overcoming significant challenges in digital health: moving from "nice to have" to mandatory systems. Clin. Pract..

[bib24] Hurvitz N., Lehman H., Hershkovitz Y. (2025). A constrained disorder principle-based second-generation artificial intelligence digital medical cannabis system: a real-world data analysis. J. Public. Health Res..

[bib25] Ilan Y. (2019). Randomness in microtubule dynamics: an error that requires correction or an inherent plasticity required for normal cellular function?. Cell. Biol. Int..

[bib26] Ilan Y. (2019). Overcoming randomness does not rule out the importance of inherent randomness for functionality. J. Biosci..

[bib27] Ilan Y. (2019). Generating randomness: making the most out of disordering a false order into a real one. J. Transl. Med..

[bib28] Ilan Y. (2019). Microtubules: From understanding their dynamics to using them as potential therapeutic targets. J. Cell. Physiol..

[bib29] Ilan Y. (2019). Why targeting the microbiome is not so successful: can randomness overcome the adaptation that occurs following gut manipulation?. Clin. Exp. Gastroenterol..

[bib30] Ilan Y. (2019). beta-glycosphingolipids as mediators of both inflammation and immune tolerance: a manifestation of randomness in biological systems. Front. Immunol..

[bib31] Ilan Y. (2020). Overcoming compensatory mechanisms toward chronic drug administration to ensure long-term, sustainable beneficial effects. Mol. Ther. Methods Clin. Dev..

[bib32] Ilan Y. (2020). Advanced tailored randomness: a novel approach for improving the efficacy of biological systems. J. Comput. Biol..

[bib33] Ilan Y. (2020). Second-Generation Digital Health Platforms: Placing the Patient at the Center and Focusing on Clinical Outcomes. Front. Digit. Health.

[bib34] Ilan Y. (2020). Order through disorder: the characteristic variability of systems. Front. Cell. Dev. Biol..

[bib35] Ilan Y. (2021). Improving global healthcare and reducing costs using second-generation artificial intelligence-based digital pills: a market disruptor. Int. J. Environ. Res. Public. Health.

[bib36] Ilan Y. (2021). Digital Medical Cannabis as Market Differentiator: Second-Generation Artificial Intelligence Systems to Improve Response. Front. Med. (Lausanne).

[bib37] Ilan Y. (2022). The constrained disorder principle defines living organisms and provides a method for correcting disturbed biological systems. Comput. Struct. Biotechnol. J..

[bib38] Ilan Y. (2022). Next-generation personalized medicine: implementation of variability patterns for overcoming drug resistance in chronic diseases. J. Pers. Med..

[bib39] Ilan Y.. The constrained disorder principle defines living organisms and provides a method for correcting disturbed biological systems—computational and Structural Biotechnology Journal 2022b;20:6087-6096. 10.1016/j.csbj.2022.11.015 PMC9668647 36420157

[bib40] Ilan Y. (2024). The co-piloting model for using artificial intelligence systems in medicine: implementing the constrained-disorder-principle-based second-generation system. Bioengineering.

[bib41] Ilan Y., Spigelman Z. (2020). Establishing patient-tailored variability-based paradigms for anti-cancer therapy: Using the inherent trajectories which underlie cancer for overcoming drug resistance. Cancer Treat. Res. Commun..

[bib42] Ilan-Ber T., Ilan Y. (2019). The role of microtubules in the immune system and as potential targets for gut-based immunotherapy. Mol. Immunol..

[bib43] Isahy Y., Ilan Y. (2021). Improving the long-term response to antidepressants by establishing an individualized platform based on variability and chronotherapy. Int. J. Clin. Pharmacol. Ther..

[bib44] Ishay Y., Kolben Y., Kessler A. (2021). Role of circadian rhythm and autonomic nervous system in liver function: a hypothetical basis for improving the management of hepatic encephalopathy. Am. J. Physiol. Gastrointest. Liver Physiol..

[bib45] Ishay Y., Potruch A., Schwartz A. (2021). A digital health platform for assisting the diagnosis and monitoring of COVID-19 progression: an adjuvant approach for augmenting the antiviral response and mitigating the immune-mediated target organ damage. Biomed. Pharmacother..

[bib46] Kalden J.R., Schulze-Koops H. (2017). Immunogenicity and loss of response to TNF inhibitors: implications for rheumatoid arthritis treatment. Nat. Rev. Rheumatol..

[bib47] Kenig A., Ilan Y. (2019). A personalized signature and chronotherapy-based platform for improving the efficacy of sepsis treatment. Front. Physiol..

[bib48] Kenig A., Kolben Y., Asleh R. (2021). Improving diuretic response in heart failure by implementing a patient-tailored variability and chronotherapy-guided algorithm. Front. Cardiovasc. Med..

[bib49] Kenig A., Kolben Y., Asleh R. (2021). Improving diuretic response in heart failure by implementing a patient-tailored variability and chronotherapy-guided algorithm. Front. Cardiovasc. Med..

[bib50] Kessler A., Weksler-Zangen S., Ilan Y. (2020). Role of the immune system and the circadian rhythm in the pathogenesis of chronic pancreatitis: establishing a personalized signature for improving the effect of immunotherapies for chronic pancreatitis. Pancreas.

[bib51] Khoury T., Ilan Y. (2019). Introducing patterns of variability for overcoming compensatory adaptation of the immune system to immunomodulatory agents: a novel method for improving clinical response to anti-TNF therapies. Front. Immunol..

[bib52] Khoury T., Ilan Y. (2019). Introducing patterns of variability for overcoming compensatory adaptation of the immune system to immunomodulatory agents: a novel method for improving clinical response to anti-TNF therapies. Front. Immunol..

[bib53] Khoury T., Ilan Y. (2021). Platform introducing individually tailored variability in nerve stimulations and dietary regimen to prevent weight regain following weight loss in patients with obesity. Obes. Res. Clin. Pract..

[bib54] Kolben Y., Weksler-Zangen S., Ilan Y. (2021). Adropin as a potential mediator of the metabolic system-autonomic nervous system-chronobiology axis: Implementing a personalized signature-based platform for chronotherapy. Obes. Rev..

[bib55] Lanzillo R., Moccia M., Palladino R. (2020). Clinical predictors of Dimethyl Fumarate response in multiple sclerosis: a real-life multicentre study. Mult. Scler. Relat. Disord..

[bib56] Miclea A., Leussink V.I., Hartung H.P. (2016). Safety and efficacy of dimethyl fumarate in multiple sclerosis: a multi-center observational study. J. Neurol..

[bib57] Mietus J.E., Peng C.K., Henry I. (2002). The pNNx files: re-examining a widely used heart rate variability measure. Heart.

[bib58] Montes Diaz G., Hupperts R., Fraussen J. (2018). Dimethyl fumarate treatment in multiple sclerosis: recent advances in clinical and immunological studies. Autoimmun. Rev..

[bib59] Navaneethakrishna M., Manuskandan S.R. (2021). Analysis of heart rate variability in normal and diabetic ECG signals using fragmentation approach. Annu. Int. Conf. IEEE Eng. Med. Biol. Soc..

[bib60] Neuhaus O., Archelos J.J., Hartung H.-P. (2003). Immunomodulation in multiple sclerosis: from immunosuppression to neuroprotection. Trends Pharmacol. Sci..

[bib61] Oleen-Burkey M., Castelli-Haley J., Lage M.J. (2012). Burden of a multiple sclerosis relapse. Patient - Patient-Cent. Outcomes Res..

[bib62] Potruch A., Khoury S.T., Ilan Y. (2020). The role of chronobiology in drug-resistance epilepsy: the potential use of a variability and chronotherapy-based individualized platform for improving the response to anti-seizure drugs. Seizure.

[bib63] Saha S., Baumert M. (2019). Intra- and inter-subject variability in EEG-based sensorimotor brain computer interface: a review. Front. Comput. Neurosci..

[bib64] Schutte A.E., Kollias A., Stergiou G.S. (2022). Blood pressure and its variability: classic and novel measurement techniques. Nat. Rev. Cardiol..

[bib65] Sen J., McGill D. (2018). Fractal analysis of heart rate variability as a predictor of mortality: A systematic review and meta-analysis. Chaos.

[bib66] Shabat Y., Lichtenstein Y., Ilan Y. (2021). Short-term cohousing of sick with healthy or treated mice alleviates the inflammatory response and liver damage. Inflammation.

[bib67] Sigawi T., Gelman R., Maimon O. (2024). Improving the response to lenvatinib in partial responders using a COnstrained-disorder-principle-based second-generation artificial intelligence-therapeutic regimen: a proof-of-concept open-labeled clinical trial. Front. Oncol..

[bib68] Sigawi T., Ilan Y. (2023). Using constrained-disorder principle-based systems to improve the performance of digital twins in biological systems. Biomimetics.

[bib69] Sigawi T., Lehmann H., Hurvitz N. (2023). Constrained disorder principle-based second-generation algorithms implement quantified variability signatures to improve the function of complex systems. J. Bioinforma. Syst. Biol..

[bib70] Souto A., Maneiro J.R., Gomez-Reino J.J. (2016). Rate of discontinuation and drug survival of biologic therapies in rheumatoid arthritis: a systematic review and meta-analysis of drug registries and health care databases. Rheumatology.

[bib71] Yang J.H., Rempe T., Whitmire N. (2022). Therapeutic advances in multiple sclerosis. Front. Neurol..

